# Optimal Defense Theory 2.0: tissue-specific stress defense prioritization as an extra layer of complexity

**DOI:** 10.1080/19420889.2019.1625661

**Published:** 2019-06-10

**Authors:** Katarzyna Wiktoria Wolinska, Matthias Leonhard Berens

**Affiliations:** Department of Plant Microbe Interactions, Max Planck Institute for Plant Breeding Research, Cologne, Germany

**Keywords:** Combined stress, abiotic stress tolerance, plant immunity, hormonal crosstalk, plant development

## Abstract

In nature, plants need to be able to quickly adapt to changing environments during their lifetime in order to maintain fitness. Different defense responses are not only costly, but often also antagonistic to one another. Hence, when faced with multiple stresses simultaneously, plants likely have to prioritize their defense responses. This type of crosstalk between different stress response pathways is suggested to balance the high costs of triggering and maintaining stress responses with the limited amount of resources available to a plant. This assumption is in accordance with the optimal defense theory (ODT), which states that living organisms put more resources into protection of the most valuable tissues, but does not explain how plants survive combined stress conditions in nature. In this review, we describe recent evidence that expands on the framework of the ODT by suggesting that under combined stress plants spatially separate contrasting stress responses, rather than protecting the most valuable tissues to simultaneously protect themselves from contrasting stressors. We discuss the implications of these findings for understanding plant responses to combined stresses and suggest potentially fruitful avenues for further research.

## Hormone crosstalk in plant defense signaling during combined stress

Plants in nature must frequently deal with changes to their surrounding environment that result in suboptimal conditions during their lifetime [1]. Critical changes in a plant’s abiotic and biotic environment must be sensed and translated into molecular, biochemical, and physiological responses that allow plants to adapt to such stressful environments in order to survive and successfully reproduce [2,3]. The presence of (potentially) pathogenic microbes triggers multiple layers of the plant’s sophisticated immune system [4]. Similarly, abiotic stresses lead to immediate molecular and physiological changes, such as gene expression changes and stomatal closure [5], the accumulation of osmo-protectants [6], as well as shortening the vegetative phase, by transition from vegetative to reproductive states [7]. Interestingly, the response pathways that protect plants against different stresses are often highly specific and can potentially antagonize the response to other types of stress [8,9]. One prominent example is the dampened immune response of plants responding to abiotic stresses, mediated by one of the phytohormones, abscisic acid (ABA) [10]. Phytohormones have been identified as important regulators in plant stress crosstalk [11]. For instance, the antagonism between abiotic and biotic stress responses is regulated through crosstalk between the abiotic stress-related phytohormone ABA and the immune-related phytohormone salicylic acid (SA) [12]. Another classic example of signaling crosstalk is the antagonism between immune-related jasmonic acid (JA) signaling and the SA pathways activated when pathogens with contrasting lifestyles attack a plant simultaneously [13]. Trade-offs between different stress response pathways are suggested to balance the high costs of triggering and maintaining stress responses with the limited amount of resources available to the plant [11,14]. The impact of hormonal crosstalk on plant fitness in the presence of multiple combined stresses remains obscure. While both antagonistic and synergistic responses have been reported [8,15], plants exhibiting antagonistic hormonal interactions do not necessarily suffer from reduced fitness under sequential treatment with drought, or biotrophic and necrotrophic pathogens [16,17].

## Developmental control of plant stress responses and the ODT

In recent years, the interaction between plant development and plant stress responses on both physiological and molecular levels and its potential role in mediating plant stress signaling crosstalk has come under increasing focus [18]. For instance, it has been demonstrated that single abiotic or biotic stresses are now known to trigger stronger responses in young compared to old leaves [19–21]. The magnitude of defense responses triggered by SA or JA is determined by the developmental age of the plant [22,23], which is coordinated through crosstalk between defense hormones and key developmental regulators, such as SHORT VEGETATIVE PHASE (SVP) MADS-domain transcription factor or the conserved developmental controller micro RNA, miR156 [22,23]. Importantly, understanding the molecular crosstalk between stress responses and plant development could solve problems we are facing in agriculture where enhancing domestication traits in crops may negatively affect plant crosstalk and thus lower plants tolerance to stresses [24]. For instance, Campos et al. [25] demonstrated how biotechnology can be used to glean molecular insights that can disentangle a well-known immunity growth crosstalk in *Arabidopsis*. Before interactions between stress responses and development were understood on a molecular level, observations of plant-herbivore interactions led to the formulation of the ODT, which states that organisms put more resources into protecting their most valuable tissues to increase fitness [26]. However, it has proven difficult to predict which tissue is the most valuable. In the case of plants, several studies have attempted to experimentally identify high-priority tissues, such as, for example, the flowers as carriers of the reproductive potential [27] or the stem, which is a crucial structural element ensuring elevation of the leaves towards the sunlight, while ensuring exchange of resources between below- and above-ground plant parts. In the context of resource allocation, young, developing leaves play an important role: they are able to more dynamically adjust to light conditions, they are better protected from photodamage under high-intensity light conditions [28] and they exhibit higher photosynthetic potential than older leaves. Hence, young leaves supply the plant with resources for a longer period of time than older, senescing leaves. Consequently, the ODT predicts that stress responses are more highly prioritized in young leaves compared to older ones, a phenomenon that has been described in natural plant – herbivore interactions [27]. However, even senescing leaves play an important role as resource re-allocation organs in plant development [29] and immunity [30]. That is why, despite the growing interest in understanding the ODT in plants during single biotic or abiotic stresses, the question of how the plant can evaluate which tissues are most valuable at a given time remains unanswered.

## Tissue-specific stress response prioritization as an additional factor in the ODT

**Figure 1.**
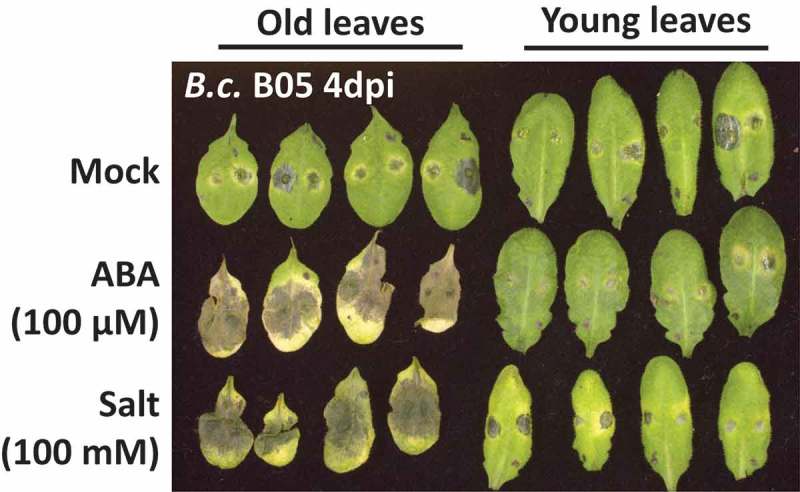
Impact of leaf age-dependent stress signaling crosstalk on leaf immune responses against *Botrytis cinerea* strain B05 (*B.c*. B05, 2 *μ*L of 2.5 × 10^5^ spores mL^−1^) in *Arabidopsis* Col-0 plants treated with control (water), 200 µM ABA or 100 mM salt. Pictures were taken four days after pathogen infection (4dpi) .

**Figure 2.**
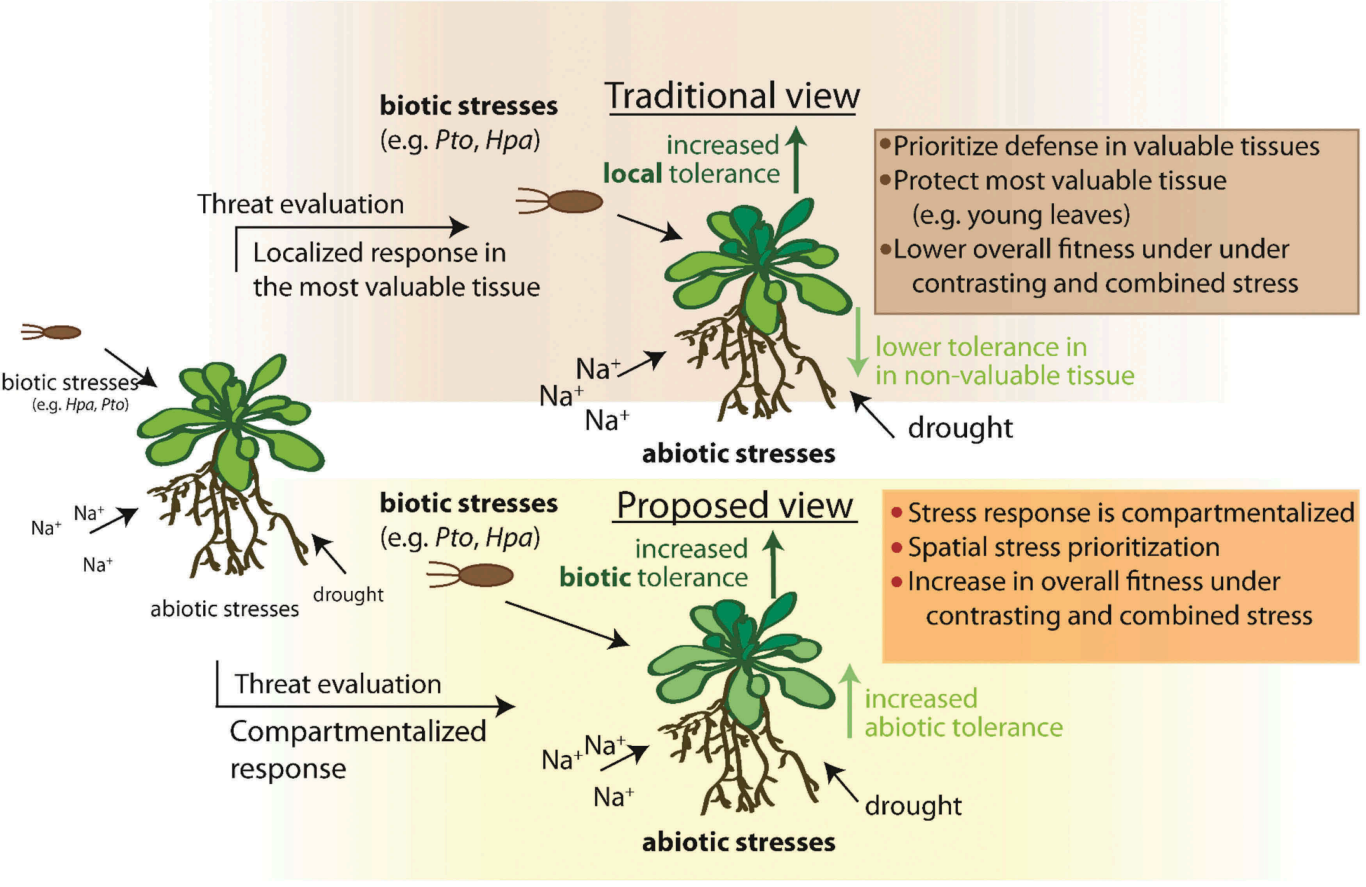
Schematic representation and comparison of the traditional view and our updated model of plant stress responses under the assumptions of the ODT. In the traditional view, hormonal crosstalk during combined stresses could prevent protection of only the most valuable parts of the plant. In contrast, in our proposed model, during combined stress, plant fitness increases due to a differential prioritization of contrasting hormonal defense responses in leaves of different ages. Na^+^ = salt stress, *Hpa* = *Hyaloperonospora arabidopsidis, Pto* = *Pseudomonas syringae pv. tomato.*

The ODT also does not explain how plants survive under combined but contrasting stresses. Optimal defense against one stress would render the plant susceptible against the other stress, which should ultimately cause a loss of fitness [16]. Recently we revealed a leaf age-dependent mechanism that plants engage to prioritize contrasting stress responses to ensure increased vegetative and reproductive fitness of the whole organism under combined stress conditions [31]. Using genetic analysis, we found that signaling components of ABA and SA hormonal crosstalk coordinate this prioritization. ABA responses suppressed immune responses only in older leaves, whereas SA signaling suppressed the antagonistic effect of ABA on immunity in young leaves. Consequently, during combined abiotic and pathogen stress, abiotic stress tolerance responses were prioritized in older leaves, while young leaves prioritized pathogen defenses [31]. Figure 1 illustrates leaf-age dependent stress responses in *Arabidopsis* plants infected with the fungal pathogen *Botrytis cinerea* in combination with either mock, ABA, or salt stress treatment. Importantly, plants that were lacking a functional PBS3 gene, a key player in SA biosynthesis and SA-mediated immunity [32,33], lost the ability to differentially prioritize contrasting stress responses, which resulted in an overall decrease in plant fitness under combined stress [31]. Our study thereby not only demonstrated that phytohormone signaling pathways play an essential role in regulating fitness under combined stress, but also described a mechanism for prioritization of defense responses, in accordance with the ODT. However, we also build on the ODT by suggesting that under combined stress, rather than protecting a more valuable tissue, plants compartmentalize stress responses into different tissues to fence off contrasting stressors, which might otherwise engage in crosstalk at downstream signaling levels (Figure 2).

## What is the signal that regulates tissue-specific stress response prioritization?

Our genetic analysis showed that leaf age rather than juvenile-to-adult development transitions in leaves regulates stress crosstalk prioritization [31]. However, the molecular signal that ensures contrasting stress responses in old and young leaves in the presence of combined stresses has not yet been identified. Previous studies have already presented experimental techniques to study tissue specificity of stress defense responses by tissue- and cell-specific metabolomics and transcriptomics analysis [34–36]. Although most of these studies were in the context of a single stress, some studies analyzed the effects of combined stress [17]. While such studies once more highlighted the importance of phytohormones in combined stress signaling, our own work suggested that additional signaling molecules might be involved in stress response prioritization: First, global analysis of hormone concentration dynamics under combined stress did not reveal strong treatment × leaf age × genotype-dependent changes for most measured hormones. Second, we found that even when the plant immune system was genetically compromised (for example in *pbs3, npr1*, and *ics1* mutants), ABA treatment further increased leaf susceptibility to biotic stress [31]. Given the overlapping roles of sugars in plant development [37], immunity [38], and abiotic stress tolerance [39], we hypothesize that sugars are promising candidate molecules as potential mediators of tissue-specific stress response prioritization. This notion is further based on the observations that, on the one hand, soluble sugar levels rise under abiotic stress where they have a role as osmoprotectants and sugar signaling is closely connected to ABA signaling [39], while on the other hand pathogens feed on soluble sugars, which are therefore an important battleground in plant immunity [38]. Reactive oxygen species (ROS) are a further class of molecules that could plausibly be hypothesized to play a role in leaf-age dependent stress response prioritization. Besides their general role in plant development [40], immunity [41,42], and abiotic stress tolerance signaling [43], ROS levels follow a leaf-age dependent trend [44]. Interestingly, Yuan et al. [45] recently demonstrated a molecular link between the defense regulators JA and SA, the growth hormone auxin, and the production of ROS as a mechanism of hormonal crosstalk. Since auxin levels are highly correlated with leaf age [31], we suggest that analyzing the interaction between auxin, SA, and ROS may yield important molecular insights into plant stress response prioritization.

## Conclusion

Plants need to constantly adapt to changing environments. While we now have a deep understanding of the molecular details of plant responses to diverse single stresses, disentangling the processes underlying defense against combined stresses has only more recently become a focus of study. We recently demonstrated that hormonal crosstalk increases fitness under combined stress by prioritizing different stress defense responses within separate leaves of a single plant. These findings, which define a role for plant developmental stage in responses to combined stresses, were in some part accordant with the ODT, which states that plants optimize defense costs by protecting only the most valuable parts of the plant. To date, however, only little is known about the molecular mechanisms that mediate the ODT under combined stresses. We identified phytohormes as essential regulators of tissue-specific combined stress defense responses, and we propose spatial sugar and ROS signaling as a promising target for future studies to understand tissue specific stress response prioritization on a molecular level.

## References

[1] Pandey P, Irulappan V, Bagavathiannan MV, et al Impact of combined abiotic and biotic stresses on plant growth and avenues for crop improvement by exploiting physio-morphological traits. Front Plant Sci. 2017 4 18;8. 10.3389/fpls.2017.00537 PMC5394115 28458674

[2] Jones JD, Dangl JL. The plant immune system. Nature. 2006 11 16;444(7117):323–329. 17108957 10.1038/nature05286

[3] Zhu JK Abiotic stress signaling and responses in plants. Cell. 2016 10 6;167(2):313–324. 27716505 10.1016/j.cell.2016.08.029 PMC5104190

[4] Mine A, Sato M, Tsuda K Toward a systems understanding of plant–microbe interactions [Review]. Front Plant Sci. 2014 8 25;5(423). 10.3389/fpls.2014.00423 PMC4142988 25202320

[5] Daszkowska-Golec A, Szarejko I Open or close the gate - stomata action under the control of phytohormones in drought stress conditions. Front Plant Sci. 2013;4:138. 23717320 10.3389/fpls.2013.00138 PMC3652521

[6] Gao J-P, Chao D-Y, Lin H-X Toward understanding molecular mechanisms of abiotic stress responses in rice. Rice. 2008 9 1;1(1):36–51.

[7] Kim W-Y, Ali Z, Park HJ, et al Release of SOS2 kinase from sequestration with GIGANTEA determines salt tolerance in Arabidopsis. Nat Commun. 2013;4:1352. 23322040 10.1038/ncomms2357

[8] Atkinson NJ, Urwin PE The interaction of plant biotic and abiotic stresses: from genes to the field. J Exp Bot. 2012 6;63(10):3523–3543. 22467407 10.1093/jxb/ers100

[9] Nguyen D, Rieu I, Mariani C, et al How plants handle multiple stresses: hormonal interactions underlying responses to abiotic stress and insect herbivory. Plant Mol Biol. 2016;91(6):727–740. 27095445 10.1007/s11103-016-0481-8 PMC4932144

[10] Bostock RM, Pye MF, Roubtsova TV Predisposition in plant disease: exploiting the nexus in abiotic and biotic stress perception and response. Annu Rev Phytopathol. 2014;52(1):517–549. 25001451 10.1146/annurev-phyto-081211-172902

[11] Berens ML, Berry HM, Mine A, et al Evolution of hormone signaling networks in plant defense. Annu Rev Phytopathol. 2017;55(1):401–425. 28645231 10.1146/annurev-phyto-080516-035544

[12] Lievens L, Pollier J, Goossens A, et al Abscisic acid as pathogen effector and immune regulator [Review]. Front Plant Sci. 2017;8(587). 10.3389/fpls.2017.00587 PMC5395610 28469630

[13] Thaler JS, Humphrey PT, Whiteman NK Evolution of jasmonate and salicylate signal crosstalk. Trends Plant Sci. 2012;17(5):260–270. 22498450 10.1016/j.tplants.2012.02.010

[14] Spoel SH, Dong X Making sense of hormone crosstalk during plant immune responses. Cell Host Microbe. 2008;3(6):348–351. 18541211 10.1016/j.chom.2008.05.009

[15] Bai Y, Kissoudis C, Yan Z, et al Plant behaviour under combined stress: tomato responses to combined salinity and pathogen stress. Plant J. 2018;93(4):781–793. 29237240 10.1111/tpj.13800

[16] Vos IA, Moritz L, Pieterse CMJ, et al Impact of hormonal crosstalk on plant resistance and fitness under multi-attacker conditions [Original Research]. Front Plant Sci. 2015;6(639). 10.3389/fpls.2015.00639 PMC4538242 26347758

[17] Coolen S, Proietti S, Hickman R, et al Transcriptome dynamics of Arabidopsis during sequential biotic and abiotic stresses. Plant J. 2016;86(3):249–267. 26991768 10.1111/tpj.13167

[18] Chandran D, Inada N, Hather G, et al Laser microdissection of Arabidopsis cells at the powdery mildew infection site reveals site-specific processes and regulators. Proc Natl Acad Sci U S A. 2010;107(1):460–465. 20018666 10.1073/pnas.0912492107 PMC2806765

[19] Moustaka J, Tanou G, Adamakis I-D, et al Leaf age-dependent photoprotective and antioxidative response mechanisms to paraquat-induced oxidative stress in arabidopsis thaliana. Int J Mol Sci. 2015;16:6. 10.3390/ijms160613989 PMC4490535 26096005

[20] Zeier J Age-dependent variations of local and systemic defence responses in Arabidopsis leaves towards an avirulent strain of Pseudomonas syringae. Physiol Mol Plant Pathol. 2005;66(1):30–39.

[21] Matsuo M, Oelmüller R REDOX RESPONSIVE TRANSCRIPTION FACTOR1 is involved in age-dependent and systemic stress signaling. Plant Signal Behav. 2015;10(11):e1051279. 26479402 10.1080/15592324.2015.1051279 PMC4883954

[22] Mao Y-B, Liu Y-Q, Chen D-Y, et al Jasmonate response decay and defense metabolite accumulation contributes to age-regulated dynamics of plant insect resistance. Nat Commun. 2017;8:13925. 28067238 10.1038/ncomms13925 PMC5233801

[23] Wilson DC, Kempthorne CJ, Carella P, et al Age-related resistance in arabidopsis thaliana involves the MADS-domain transcription factor SHORT VEGETATIVE PHASE and direct action of salicylic acid on pseudomonas syringae. Mol Plant Microbe Interact. 2017 11;30(11):919–929. 28812948 10.1094/MPMI-07-17-0172-R

[24] Shigenaga AM, Berens ML, Tsuda K, et al Towards engineering of hormonal crosstalk in plant immunity. Curr Opin Plant Biol. 2017;38:164–172. 28624670 10.1016/j.pbi.2017.04.021

[25] Campos ML, Yoshida Y, Major IT, et al Rewiring of jasmonate and phytochrome B signalling uncouples plant growth-defense tradeoffs. Nat Commun. 2016 8;30(7):12570. 10.1038/ncomms12570 PMC5155487 27573094

[26] Meldau S, Erb M, Baldwin IT Defence on demand: mechanisms behind optimal defence patterns. Ann Bot. 2012 12;110(8):1503–1514. 23022676 10.1093/aob/mcs212 PMC3503495

[27] Keith RA, Mitchell-Olds T Testing the optimal defense hypothesis in nature: variation for glucosinolate profiles within plants. PLoS One. 2017;12(7):e0180971. 28732049 10.1371/journal.pone.0180971 PMC5521783

[28] Bielczynski LW, Lacki MK, Hoefnagels I, et al Leaf and plant age affects photosynthetic performance and photoprotective capacity. Plant Physiol. 2017 12;175(4):1634–1648. 29018097 10.1104/pp.17.00904 PMC5717728

[29] Thomas H Senescence, ageing and death of the whole plant. New Phytol. 2013 2;197(3):696–711. 23176101 10.1111/nph.12047

[30] Haffner E, Konietzki S, Diederichsen E Keeping control: the role of senescence and development in plant pathogenesis and defense. Plants (Basel). 2015 7 13;4(3):449–488. 27135337 10.3390/plants4030449 PMC4844401

[31] Berens ML, Wolinska KW, Spaepen S, et al Balancing trade-offs between biotic and abiotic stress responses through leaf age-dependent variation in stress hormone cross-talk. Proc Nat Acad Sci. 2019;116(6):2364–2373. 30674663 10.1073/pnas.1817233116 PMC6369802

[32] Torrens-Spence MP, Bobokalonova A, Carballo V, et al. PBS3 and EPS1 complete salicylic acid biosynthesis from isochorismate in Arabidopsis Biorxiv. 2019:601948. 10.1016/j.molp.2019.11.005 31760159

[33] Rekhter D, Mohnike L, Feussner K, et al. Enhanced Disease Susceptibility 5 (EDS5) is required for N-hydroxy pipecolic acid formation. Biorxiv. 2019:630723.

[34] Wang Y, Jiao Y Advances in plant cell type-specific genome-wide studies of gene expression. Front Biol. 2011;6(5):384.

[35] Li D, Heiling S, Baldwin IT, et al Illuminating a plant’s tissue-specific metabolic diversity using computational metabolomics and information theory. Proc Nat Acad Sci. 2016 113(47):E7610–E7618. 27821729 10.1073/pnas.1610218113 PMC5127351

[36] Giacomello S, Salmen F, Terebieniec BK, et al Spatially resolved transcriptome profiling in model plant species. Nat Plants. 2017 5;8(3):17061. 10.1038/nplants.2017.61 28481330

[37] Sakr S, Wang M, Dedaldechamp F, et al The sugar-signaling hub: overview of regulators and interaction with the hormonal and metabolic network. Int J Mol Sci. 2018;19:9. 10.3390/ijms19092506 PMC6165531 30149541

[38] Yamada K, Saijo Y, Nakagami H, et al Regulation of sugar transporter activity for antibacterial defense in Arabidopsis. Science. 2016 12 16;354(6318):1427–1430. 27884939 10.1126/science.aah5692

[39] Rosa M, Prado C, Podazza G, et al Soluble sugars–metabolism, sensing and abiotic stress: a complex network in the life of plants. Plant Signal Behav. 2009;4(5):388–393. 19816104 10.4161/psb.4.5.8294 PMC2676748

[40] Mhamdi A, Van Breusegem F Reactive oxygen species in plant development. Development. 2018 8 9;145(15). 10.1242/dev.164376 30093413

[41] Kadota Y, Shirasu K, Zipfel C Regulation of the NADPH oxidase RBOHD during plant immunity. Plant Cell Physiol. 2015 8;56(8):1472–1480. 25941234 10.1093/pcp/pcv063

[42] Qi J, Wang J, Gong Z, et al Apoplastic ROS signaling in plant immunity. Curr Opin Plant Biol. 2017;38:92–100. 28511115 10.1016/j.pbi.2017.04.022

[43] Choudhury FK, Rivero RM, Blumwald E, et al Reactive oxygen species, abiotic stress and stress combination. Plant J. 2017 6;90(5):856–867. 27801967 10.1111/tpj.13299

[44] Srivalli S, Khanna-Chopra R Delayed wheat flag leaf senescence due to removal of spikelets is associated with increased activities of leaf antioxidant enzymes, reduced glutathione/oxidized glutathione ratio and oxidative damage to mitochondrial proteins. Plant Physiol Biochem. 2009;47(8):663–670. 19394842 10.1016/j.plaphy.2009.03.015

[45] Yuan H-M, Liu W-C, Lu Y-T CATALASE2 coordinates SA-mediated repression of both auxin accumulation and JA biosynthesis in plant defenses. Cell Host Microbe. 2017;21(2):143–155. 28182949 10.1016/j.chom.2017.01.007

